# CD40 mutant expression and its clinical significance to prognosis in gastric cancer patients

**DOI:** 10.1186/1477-7819-12-167

**Published:** 2014-05-28

**Authors:** Wei-Qing Zhao, Xiao-Dong Li, Hong-Bing Shi, Jun Wu, Jie-Min Zhao, Mei Ji, Chang-Ping Wu

**Affiliations:** 1 Department of Oncology, The Third Affiliated Hospital of Soochow University, 185 Juqian Street, Changzhou, Jiangsu Province, People’s Republic of China; 2 Department of Tumor Biological Treatment, The Third Affiliated Hospital of Soochow University, 185 Juqian Street, Changzhou, Jiangsu Province, People’s Republic of China

**Keywords:** Gastric cancer, CD40 mutant, Immunohistochemistry

## Abstract

**Background:**

We aimed to detect CD40 mutant expression and evaluate its clinical significance in gastric cancer.

**Methods:**

CD40 mutant expression in 78 cases of gastric cancer tissues, 10 cases of normal gastric tissues, and 10 cases of gastric adenoma tissues by immunohistochemical test. Survival analyses were also performed.

**Results:**

The positive CD40 mutant rate in gastric cancer was 55.1% (43/78). No positive CD40 mutant staining was observed in the normal gastric tissue or the gastric adenoma. CD40 mutants expression was significantly correlated with invasive depth, lymph metastasis, and TNM stage (*P* <0.05). Cases with negative CD40 mutant expression had a significantly longer median survival time than those with positive CD40 mutant expression (40 *vs.* 14 months, *P* <0.05). A lower death risk in negative CD40 mutant cases was observed comparing with positive CD40 mutant cases.

**Conclusions:**

Positive CD40 mutant expression suggests a poorer prognosis of gastric cancer cases.

## Background

Gastric cancer is the second leading cause of cancer-related death worldwide, with the highest incidence in Eastern Asian and Eastern European countries [1-3]. It was estimated that 989,000 new cases and 738,000 deaths had occurred worldwide in 2008 alone, which accounted for 8% of the total new cases and 10% of the total cancer-related deaths [3].

With the advances in molecular biology, much progress has been achieved in our understanding of the molecular pathogenesis of cancer. CD40 is a cell surface receptor that belongs to the tumor necrosis factor-receptor superfamily, originally identified in T cells. CD40 was first identified and functionally characterized on B lymphocytes [4,5]. Later reports revealed that the expression of CD40 was not restricted to B cells but that it was also expressed on monocytes, dendriticcell, and on non-hematopoietic cells including keratinocytes, fibroblasts, neurons, endothelial, epithelial, and tumor cells.

CD40 is a crucial member of the group of co-stimulating molecules, orchestrating both humoral and cell-mediated immune responses, and is closely related with tumor invasion and metastasis [5,6]. CD40 provides a conduit through which the immune system can suppress growth and induce apoptosis of CD40-expressing epithelial cancers [7].

CD40 mutant (CAC → CAA, 78His → 78Gln, NCBI Assay ID: ss23134804, Reference SNP ID: rs17177493) is a variant type of CD40 found on the surface of the U266 cell line and in freshly isolated tumor cells. The mutated residue of this mutant locates in a region which is important for binding to CD40L. CD40 mutant is translocated to the CD40 signalosome and involved in CD40 signal transduction. It has been reported that CD40 mutant alters partial epitope of CD40 and has a different ability to combine with antibodies combining with wild-type CD40, indicating that CD40 mutant might play an important role in tumor development. CD40 mutant can also be considered as a possible target for anti-cancer immunotherapy [8]. However, the mechanisms of the multifaceted roles of CD40 in gastric cancer are still not completely understood.

The present study aimed to examine the CD40 mutant expression in gastric cancer tissues and investigate the correlation of CD40 mutant expression and clinical outcome.

## Methods

### Patient enrollment

A total of 78 gastric cancer patients diagnosed as gastric cancer by after-operation pathological examinations were enrolled between 1 January 2002 and 31 December 2005 in The Third Affiliated Hospital of Soochow University in China. No patients received radiotherapy or chemotherapy before the enrollment. Moreover, those who had secondary malignancies were excluded. Of the 78 cases, 58 were men and 20 were women (age range, 28 to 77 years; average age, 55 years). From 2004 to 2005, the 10 cases of normal gastric tissue, with four cases of male and six cases of female normal gastric tissue (age range, 51 to 77 years; average age, 58 years), and 10 cases of gastric adenoma (age range, 55 to 67 years; average age, 60 years), were obtained from outpatients. The 78 cases consisted of 43 cases of well/moderate differentiation types (papillary adenocarcinoma, well-differentiated tubular adenocarcinoma, moderately differentiated tubular adenocarcinoma) and 35 cases of poor differentiation types (poor-differentiated tubular adenocarcinoma, signet ring cell carcinoma, mucinous carcinoma) according to Kloppel criteria [9]. According to the Response Evaluation Criteria in Solid Tumors criteria (version 1.1), there were 17 cases of stage I or II, and 61 cases of stage III or IV. Every patient, enrolled in this study, signed a written informed consent form approved by the Medical Ethics Committee of Soochow University, which also approved the study protocol.

### Immunohistochemistry

Antibody to CD40 mutant protein was purchased from the Institute of Medical Biotechnology, Soochow University. Diaminobenzidine staining kit was purchased from Fuzhou Maixin Biotechnology Limited Cooperation.

Elivision method was used. For the immunohistochemical study, formalin-fixed, paraffin embedded tissue samples from all cases were obtained from patients who underwent surgeries at our hospital. For CD40 mutant immunostaining with goat polyclonal antibodies, tissue sections (5 μm) were deparaffinized in xylene and rehydrated in an ethanol series. The sections were then treated for 30 min with 0.3% hydrogen peroxide to block endogenous peroxidase activity, then subsequently washed with phosphate-buffered saline (PBS) and unmasked in citrate antigen unmasking solution in an autoclave for 20 min at 120°C. The sections were incubated with goat serum for 15 min at room temperature and then were incubated with the primary antibodies for 1 h at room temperature. The bound primary antibodies were detected by adding anti-mouse secondary antibodies and avidin/biotin/horseradish peroxidase complex for 30 min at room temperature. The sections were visualized using solid diaminobenzine diluted in PBS, counterstained with Mayer’s hematoxylin, and finally mounted. After that, they were incubated with HRP-labeled anti-mouse immunoglobulin G as the secondary antibody. Substrate chromogen was added and the specimens were counterstained with hematoxylin.

Three independent investigators assessed CD40 mutant positivity semi-quantitatively without prior knowledge of the clinical follow-up data. Every slice was observed for representative visual fields randomly. Brown granules as considered as positive CD40 mutant staining mainly on the cytoplasmic membrane, and it was also considered as positive staining while cytoplasm was stained. The intensity of cytoplasmic staining was graded into two easily reproducible subgroups: a. negative (−): the percentage of CD40 mutant positive cells <25% or no detectable coloration; b. positive (+): ≥25%.

### Statistical analysis

All statistical analyses were performed using SPSS 13.0 software (SPSS, Inc.). Correlations between clinicopathologic variables and CD40 mutant levels were compared by the rank sum tests (two-group analysis using the Wilcoxon test and multigroups analysis using the Kruskal-Wallis test). Chi-square test was used to compare the categorical data. COX model was used to evaluate the strength of the association between CD40 mutant expression and death. Kaplan-Meier method was used to calculate the overall survival time (OST). Log-rank test was used to compare the OST. *P* values <0.05 were considered significant.

## Results

### CD40 mutant expression

**Figure 1:**
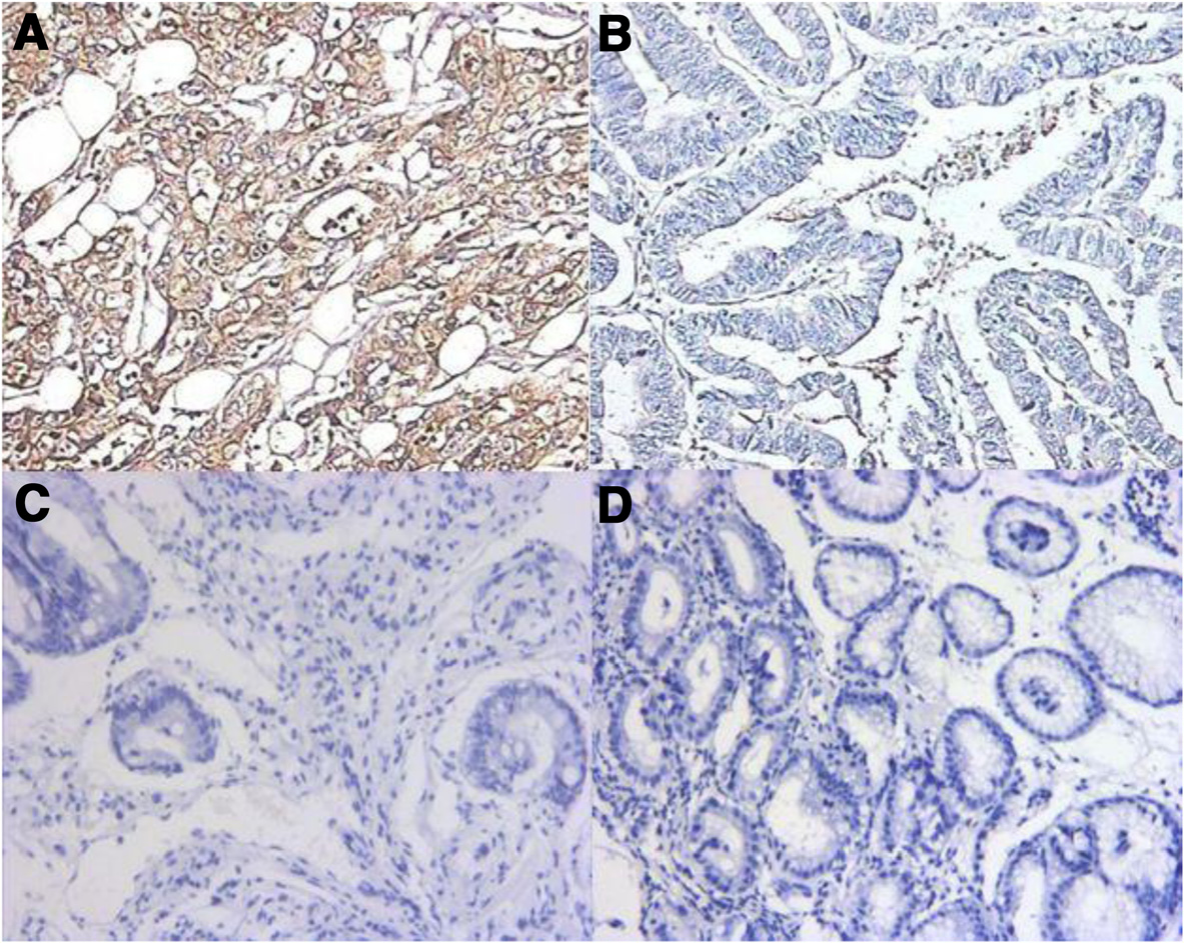
**Expression of CD40 mutant molecule in various tissue. (A)** Positive expression of CD40 mutant molecule in gastric cancer tissue (×200) **(B)** Negative expression of CD40 mutant molecule in gastric cancer tissue (×200) **(C)** Negative expression of CD40 mutant molecule in normal gastric tissue (×200) **(D)** Negative expression of CD40 mutant molecule in gastric adenoma tissue (×200).

The positive CD40 mutant rate in gastric cancer was 55.1% (43/78). No positive CD40 mutant staining was observed in the 10 cases of normal gastric tissue or the 10 cases of gastric adenoma (Figure 1).

### Association between CD40 mutant expression and clinicopathological characteristics

The positive rate of CD40 mutant expression: (1) in cases with tumor invasion to deep muscle layer was significantly higher than that in cases with tumor invasion not to deep muscle layer (61.8% *vs.* 10%, *χ*^*2*^ = 9.444, *P* = 0.002); (2) in cases with lymph node metastasis was significantly higher than that in cases without lymph node metastasis (65% *vs.* 37%, *χ*^*2*^ = 7.802, *P* = 0.005); (3) in cases of stage III and IV was significantly higher than that in cases of stages I and II (63.9% *vs.* 23.5%, *χ*^*2*^ = 8.774, *P* = 0.003). Additionally, CD40 mutant expression was not correlated with sex, age, tumor size, or pathological differentiation (*P* >0.05) (Table 1).

**Table 1:** Association between gastric cancer patients’ clinicopathological characteristics s and CD40 mutant levels in tumor tissues (n = 78)

**Characteristics**	**Cases**	**CD40 mutant expression**		***χ***^***2***^	***P***
		**Positive**	**Negative**		
Sex					
Male	58	33	25	0.286	0.593
Female	20	10	10		
Age (years)					
≥60	48	36	12	2.849	0.091
<60	30	17	13		
Tumor size (cm)					
≥5	37	20	17	0.033	0.856
<5	41	23	18		
Differentiation					
Well/moderate	43	20	23	2.074	0.15
Poor	35	22	13		
Depth of tumor invasion					
Not to deep muscle layer	10	1	9	9.444	0.002^a^
Deep muscle layer	68	42	26		
Lymph nodes metastasis					
Yes	60	39	21	7.802	0.005^a^
No	18	5	13		
TNM stage					
I and II	17	4	13	8.774	0.003^a^
III and IV	61	39	22		

^a^*P* <0.05.

### Survival analysis

**Figure 2:**
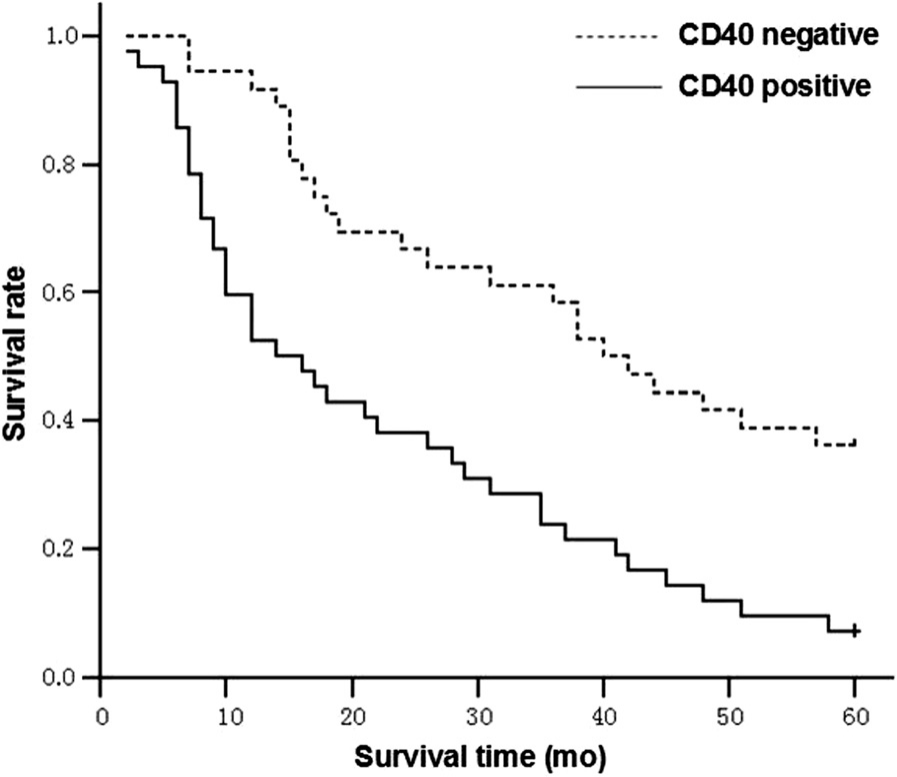
**Overall survival curves for gastric cancer patients.** The median overall survival time of cases with negative CD40 mutant expression is significantly longer than that of cases with positive negative CD40 mutant expression (40 *vs.* 14 months, 95% CI 30.6-49.4 and 6.7-21.3, respectively, *χ*^*2*^ = 14.229, *P* <0.001).

The median overall survival time of cases with negative CD40 mutant expression was significantly longer than that of cases with positive negative CD40 mutant expression (40 *vs.* 14 months, 95% CI 30.6-49.4 and 6.7-21.3, respectively, *χ*^*2*^ = 14.229, *P* <0.001) (Figure 2).

### COX model analysis

A lower death risk in negative CD40 mutant cases was observed comparing with positive CD40 mutant cases after sex, age, depth of tumor invasion, and lymph node metastasis were adjusted (HR = 0.383, 95% CI 0.227-0.646, *P* <0.001).

## Discussion

To the best of our knowledge, there have not been any reports on CD40 mutant expression in gastric cancer cases, normal gastric tissues, and gastric adenoma.

Several studies found that CD40 expression was significantly correlated with lymph node metastasis and distant metastasis as well as TNM stage, meanwhile patients with positive CD40 mutant expression had a poorer prognosis [10,11].

Our results also suggest that CD40 mutant may be an important factor influencing gastric cancer patients’ survival time, which suggests that CD40 mutant may be an independent negative-regulating molecule influencing the prognosis of gastric cancer.

Furthermore, no positive CD40 mutant staining was observed in normal gastric tissues or gastric adenoma tissues, hence there is a possibility that CD40 mutant is considered as a tumor biomarker for differentiating malignant and benign tumors. We have reason to believe that CD40 mutant level in patients’ serum can be detected to observe the correlation with that in tissues, but further studies with a larger sample size should be performed to confirm the liability.

Choudhury *et al.*[12] found that CD40 could transmit the signals which induced apoptosis. We reckon that tumor cells inhibit T lymphocytes activation by expressing CD40 mutants to escape from immune monitoring. Our results reveal that there is correlation between CD40 mutant expression and the stages of gastric cancer as well as the prognosis, implying a possibility that the specific antibody of CD40 mutant may induce the apoptosis of gastric cancer cells, which means that CD40 mutant could be considered as a new target for tumor immunotherapy and immune intervention. By using anti CD40 antibody or CD40 siRNA, this possible target could be done *in vitro* first and then confirmed on animal models before being enrolled into clinical trials.

We believe that CD40 mutant plays an important role in mediating and regulating the biological behaviors of gastric cancer cells, and moreover may be a novel target for diagnosis and therapy of cancers. However, the present preliminary study includes a great heterogenicity of the enrolled patients to evaluate the prognosis of patients with gastric cancer, and the role of CD40 mutant needs to be confirmed. The more exact functions and mechanisms require progressive studies and the clinical significance of CD40 mutant should be proved by more randomized, controlled clinical trials with larger enrolled case sizes.

## Abbreviations

OST: Overall survival time; PBS: Phosphate-buffered saline.

## Competing interests

The authors declare that they have no competing interests.

## Authors’ contributions

WQZ and XDL performed the experiments. HBS analyzed the data. JW and JMZ wrote the manuscript. WQZ and XDL helped with manuscript editing. CPW and MJ provided the study concept and guided the writing. All authors read and approved the final manuscript.

## Authors’ information

Wei-Qing Zhao and Xiao-Dong Li are the co-first authors.
